# Error and discrepancy in radiology: inevitable or avoidable?

**DOI:** 10.1007/s13244-016-0534-1

**Published:** 2016-12-07

**Authors:** Adrian P. Brady

**Affiliations:** Radiology Department, Mercy University Hospital, Cork, Ireland

**Keywords:** Radiology, Error, diagnostic, Error sources, Misdiagnosis, Quality improvement

## Abstract

**Abstract:**

Errors and discrepancies in radiology practice are uncomfortably common, with an estimated day-to-day rate of 3–5% of studies reported, and much higher rates reported in many targeted studies. Nonetheless, the meaning of the terms “error” and “discrepancy” and the relationship to medical negligence are frequently misunderstood. This review outlines the incidence of such events, the ways they can be categorized to aid understanding, and potential contributing factors, both human- and system-based. Possible strategies to minimise error are considered, along with the means of dealing with perceived underperformance when it is identified. The inevitability of imperfection is explained, while the importance of striving to minimise such imperfection is emphasised.

***Teaching Points*:**

• *Discrepancies between radiology reports and subsequent patient outcomes are not inevitably errors*.

• *Radiologist reporting performance cannot be perfect, and some errors are inevitable*.

• *Error or discrepancy in radiology reporting does not equate negligence*.

• *Radiologist errors occur for many reasons, both human- and system-derived*.

• *Strategies exist to minimise error causes and to learn from errors made*.

## Definition of error/discrepancy

It was recently estimated that one billion radiologic examinations are performed worldwide annually, most of which are interpreted by radiologists [1]. Most professional bodies would agree that all imaging procedures should include an expert radiologist’s opinion, given by means of a written report [2]. This activity constitutes much of the daily work of practising radiologists. We don’t always get it right.

Although not always appreciated by the public, or indeed by referring doctors, radiologists’ reports should not be expected to be definitive or incontrovertible. They represent clinical consultations, resulting in opinions which are conclusions arrived at after weighing of evidence [3]; **“opinion”** can be defined as *“a view held about a particular subject or point; a judgement formed; a belief”* [4]. Sometimes it is possible to be definitive in radiological diagnoses, but in most cases, radiological interpretation is heavily influenced by the clinical circumstances of the patient, relevant past history and previous imaging, and myriad other factors, including biases of which we may not be aware. Radiological studies do not come with inbuilt labels denoting the most significant abnormalities, and interpreting them is not a binary process (normal vs abnormal, cancer vs “all-clear”).

In this context, defining what constitutes radiological error is not straightforward. The use of the term **“error”** implies that there is no potential for disagreement about what is “correct”, and indicates that the reporting radiologist should have been able to make the correct diagnosis or report, but did not [3]. In real life, there is frequently room for legitimate differences of opinion about diagnoses or for “failure” to identify an abnormality that can be seen in retrospect. Expert opinion often forms the basis for deciding whether an error has been made [3], but it should be noted that **“experts”** themselves may also be subject to question (*“An expert is someone who is more than fifty miles from home, has no responsibility for implementing the advice he gives, and shows slides.”* - Ed Meese, US Attorney General 1985–88).

Any discrepancy in interpretation that deviates substantially from a consensus of one’s peers is a reasonable and commonly accepted definition of **interpretive radiological error** [1], but even this is a loose description of a complex process, and may be subject to debate in individual circumstances. Certainly, in some circumstances, diagnoses are proven by pathologic examination of surgical or autopsy material, and this proof can be used to evaluate prior radiological diagnoses [1], but this is not a common basis for determining whether error has occurred. Many cases of supposed error, in fact, fall within the realm of reasonable differences of opinions between conscientious practitioners. **“Discrepancy”** is a better term to describe what happens in many such cases.

This is not to suggest that radiological error does not occur; it does, and frequently. Just how frequently will be addressed in another section of this paper.

### Negligence

Leonard Berlin, writing in 1995, found that the rate of radiology-related malpractice lawsuits in Cook County, Illinois, USA, was rising inexorably, with the majority of suits for missed diagnosis, and we have no reason to believe that this pattern has since changed. Interestingly, his data showed a progressive reduction in the length of time between the introduction of a new imaging technology and the first filed lawsuit arising from its use, from over 10 years for ultrasound (first suit 1982), to 8 years for CT (first suit 1982), and 4 years for MRI (first suit 1987) [5].

The distinction between “acceptably” or “understandably” failing to perceive or report an abnormality on a radiological study and negligently failing to report a lesion is an important one, albeit one that is difficult to explain to laypersons or juries. As Berlin wrote:

*“[F]rom a practical point of view once an abnormality on a radiograph is pointed out and becomes so obvious that lay persons sitting as jurors can see it, it is not easy to convince them that a radiologist who is trained and paid for seeing the lesion should be exonerated for missing it. This is especially true when the missing of that lesion has delayed the timely diagnosis and the possible cure of a malignancy that is eventually fatal”* [6].

A major influence on the determination of whether an initially missed abnormality should have been identified arises in the form of **hindsight bias**, defined as the *“tendency for people with knowledge of the actual outcome of an event to believe falsely that they would have predicted the outcome”* [6]. This “creeping determinism” involves automatic and immediate integration of information about the outcome into one’s knowledge of events preceding the outcome [6]. Expert witnesses are frequently influenced by their knowledge of the outcome in determining whether a radiologist, acting reasonably, ought to have detected an abnormality when reporting a study prior to the outcome being known, and thus in suggesting whether failure to detect the abnormality constituted negligence.

Berlin quotes a Wisconsin (USA) appeals court decision which helpfully teases out some of these points:

*“In determining whether a physician was negligent, the question is not whether a reasonable physician, or an average physician, should have detected the abnormalities, but whether the physician used the degree of skill and care that a reasonable physician, or an average physician, would use in the same or similar circumstances…A radiologist may review an x-ray using the degree of care of a reasonable radiologist, but fail to detect an abnormality that, on average, would have been found… Radiologists simply cannot detect all abnormalities on all x-rays… The phenomena of “errors in perception” occur when a radiologist diligently reviews an x-ray, follow[s] all the proper procedures, and use[s] all the proper techniques, and fails to perceive an abnormality, which, in retrospect is apparent… Errors in perception by radiologists viewing x-rays occur in the absence of negligence”* [6].

Radiologists base their conclusions on a varying number of premises (e.g. available clinical information, statistical likelihood). Any of the bases for conclusions may prove to have been false. Subsequent information may show the original conclusion to have been false, but this does not constitute a *prima facie* error in judgement, and the possibility that a different radiologist might have come to a different conclusion based upon the same information does not imply negligence on its own [7].

It is important to avoid the temptation (beloved by plaintiffs’ lawyers) to apply the principle *“radiologists have a duty to interpret radiographs correctly”* to specific instances (*“radiologists have a duty to interpret this particular radiograph correctly”).* The inference that missing an abnormality on a specific radiograph automatically constitutes malpractice is not correct [7]. Experienced, competent radiologists may miss abnormalities, and may be unaware of having done so. Experienced radiologists may make different judgements based on the same study; thus differences in judgement are not negligence [7]. Unfortunately, juries are often swayed by compassion for an injured defendant, and research has shown that the results of malpractice suits are often related to the degree of disability or injury rather than to the nature of the event or whether physician negligence was present [7].

## Distribution of radiologist performance

**Fig. 1:**
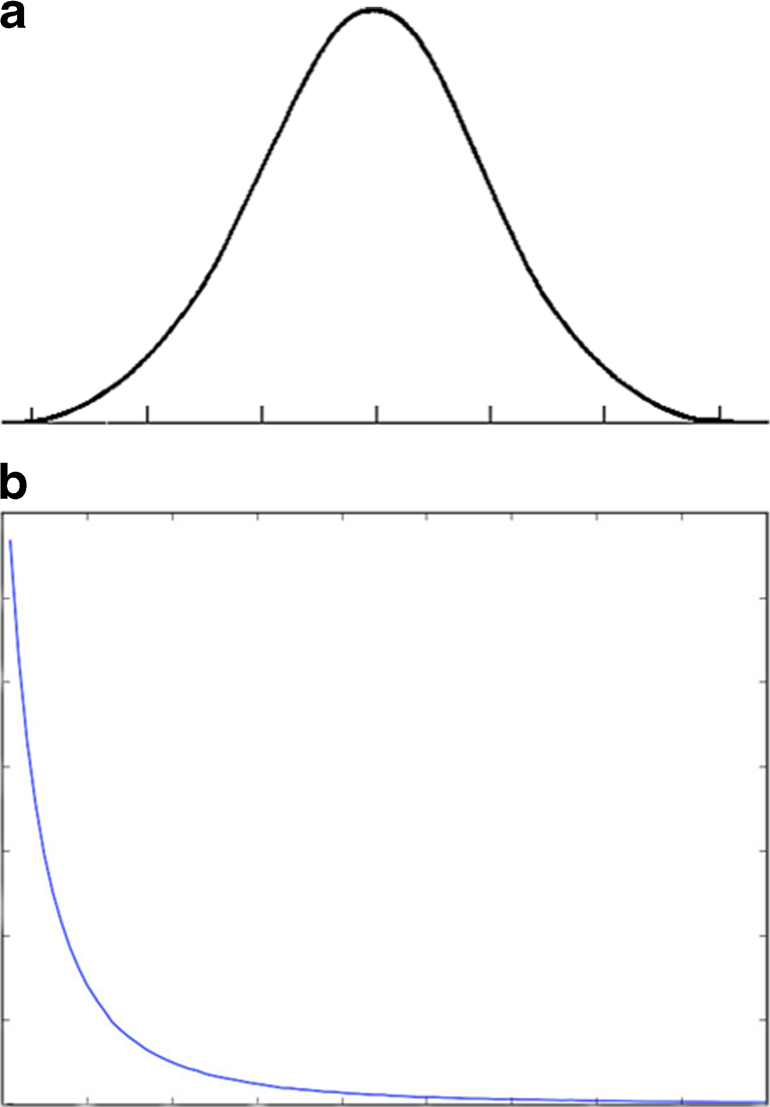
**a** Gaussian (normal) distribution. **b** Paretian (power) distribution

The American humorist Garrison Keillor reports the news from his fictional home, Lake Wobegon, on his weekly radio show, *A Prairie Home Companion*, concluding each monologue with *“That’s the news from Lake Wobegon, where all the women are strong, all the men are good looking, and all the children are above average"* [8]. Sadly, the statistical absurdity underpinning the joke is not always appreciated by media or political commentators, who often fail to appreciate the necessity of “below-average” performance. If one assumes that the accuracy of radiological performance approximates a normal (Gaussian) distribution (Fig. 1a), then about half of that performance must lie below the median—must be “below average”. That does not mean that these radiologists are substandard by definition. Inevitably, some radiological performance will fall so far to the left extreme of the distribution that it will be judged to be below acceptable standards, but the threshold defining what is acceptable performance is somewhat arbitrary, and relies upon the loose definition based on peer standards outlined in the "Definition" section of this paper.

A Gaussian distribution of performance is not necessarily universally accepted. In 2012, O’Boyle and Aguinis published a review of studies measuring performance among more than 600,000 researchers, entertainers, politicians, and amateur and professional athletes [9]. The authors found that individual performance was not normally distributed, but instead followed a Paretian (power law) distribution (Fig. 1b), such that most performance was clustered to the left side of the reverse exponential curve, and most accomplishments were achieved by a small number of super-performers. On this model, most performers are below “average”, and thus less productive and more likely to make mistakes than the super-performers, or even than the median, which is skewed towards the higher end of performance.

The subtleties and implications of these statistical concepts are often not understood—or are wilfully ignored—by media commentators or the general public, and thus the concept of “average” is often misinterpreted as the lowest acceptable standard of behaviour. As my 14-year-old son recently remarked to me, most people have an above-average number of legs.

Regardless of the shape of the curve of radiological performance, however, the aim of any quality improvement programme should be to shift the curve continually to the right [10] and, if possible, to narrow the width of the curve such that the underlying culture in the workforce is one of striving to minimise variability in performance quality and to continually improve performance in any way possible.

## How prevalent is radiologic error?

Table 1 lists a sample of published studies, ranging from 1949 to the present, which have assessed the frequency of radiological errors or discrepancies. Leonard Berlin has published extensively on this issue, and cites a real-time day-to-day radiologist error rate averaging 3–5%, and a retrospective error rate among radiologic studies averaging 30% [22]. Applying a 4% error rate to the worldwide one billion annual radiologic studies equates to about 40 million radiologist errors per annum [1].

**Table 1:** Sample of published studies of radiological error

Year	Author	Ref	Material	Key points	Comments
2001	Goddard et al.	[11]	Various	Clinically significant error rate of 2–20%, depending on radiological investigation	
1981	Forrest et al.	[12]	Retrospective review of previous chest x-rays (CXRs) in patients subsequently diagnosed with lung cancer	False-negative rate of 40%	Lesions visible but not reported on prior studies
1983	Muhm at al	[13]	Lung cancers detected by plain radiography screening	90% of cancers detected visible in retrospect on prior radiographs going back months or, in some cases, years (53 months in one case)	
1993	Harvey et al.	[14]	Review of prior mammograms in patients in whom impalpable breast cancer subsequently diagnosed by mammography	Evidence of carcinoma identifiable on prior studies in 41% when blindly reinterpreted, and in 75% when reviewers were aware of subsequent findings	
1999	Quekel et al.	[15]	Non-small cell lung cancer diagnosed on plain CXR	19% missed diagnosis rate	16-mm median diameter of missed lesions, median delay in diagnosis of 472 days
1949	In Robinson (1997)	[3]	CXR in patients with suspected TB	Interpreted differently by different observers in 10–20%	
1990, 1994	Markus et al., Brady et al.	[16, 17]	Barium enema	Average observer missed 30% of visible lesions	Supposed gold standard of colonoscopy also subject to error
1999	Robinson	[18]	Emergency dept. plain radiographs	Major disagreement between two observers in 5–9% of cases	Estimated error incidence per observer of 3–6%
1997	Tudor et al.	[19]	Plain radiographs	Mean accuracy: 77% without clinical information, 80% with clinical information. Modest improvements in sensitivity, specificity and inter-observer agreement with clinical information	Five experienced radiologists reported mix of validated normal and abnormal studies 5 months apart. No clinical information on first occasion, relevant clinical information provided on second occasion
2008	Siewert et al.	[20]	Oncologic CT	Discordant interpretations in 31–37%, with resultant change in radiological staging in 19%, and change in patient treatment in up to 23%	
2007	Briggs et al.	[21]	Neuro CT & MR	13% major & 21% minor discrepancy rates (undercalls, overcalls & misinterpretations)	Specialist neuroradiologist second reading of studies initially interpreted by general radiologists

Many of the papers quoted (and the myriad other, similar studies) describe retrospective assessment, with varying degrees of blinding at the time of re-assessment of studies. Prospective studies have also been published. A major disagreement rate of 5–9% was identified between two observers in interpreting emergency department plain radiographs, with an error incidence per observer of 3–6% [18]. A cancer misdiagnosis (false-positive) rate of up to 61% has been quoted for screening mammography [23]. In the context of 38,293,403 screening mammograms performed in the US in 2013, this rate has significant implications for patient morbidity and anxiety. Discordant interpretations of oncologic CT studies have been reported in 31–37% of cases [20].

Error or discrepancy rates can be influenced by the standard against which the initial report is measured. A 2007 study of the impact of specialist neuroradiologist second reading of CT and MR studies initially interpreted by general radiologists found a 13% major and 21% minor discrepancy rate [21].

Most of these studies are based on identification of inter-observer variation. Intra-observer variation, however, should not be ignored. A 2010 study from Massachusetts General Hospital tasked three experienced abdominal imaging radiologists with blindly re-interpreting 60 abdominal and pelvic CTs, 30 of which had previously been reported by someone else and 30 by themselves. Major inter-observer and intra-observer discrepancy rates of 26% and 32%, respectively, were found [24].

Similar reports in the literature of the last 60 years are legion; the above examples serve to show the consistency of discrepancy rates across modalities, subspecialties and time. Given these apparently constant, high discrepancy rates, it seems far-fetched to imagine that these “errors” are entirely the product of “bad radiologists”.

## Other medical specialties

Inherent in the work produced by radiologists (and histopathologists) is the fact that virtually every clinical act we perform is available for re-interpretation or review at a later date. Digital archival systems have virtually eliminated the loss of radiological material, even after many years. This has been a boon to patient care, and underpins much multidisciplinary team activity. It has also been a boon to those interested in researching radiological error and those interested in using archival data for other purposes, including litigation.

This capacity to revisit prior clinical decisions and acts is less available for most other medical specialties, and thus the literature detailing the prevalence of error in other specialties is less extensive. Nonetheless, some such data exist. A study from the Mayo Clinic published in 2000 reviewed the pre mortem clinical diagnoses and post mortem diagnoses in 100 patients who died in the medical intensive care unit [25]. In 16%, autopsies revealed major diagnoses that, if known before death, might have led to a change in therapy and prolonged survival; in another 10%, major diagnoses were found at autopsy that, if known earlier, would probably not have led to a change in therapy. Berlin quotes Harvard data showing adverse events occurring in 3.7% of hospitalisations in New York, and data from other states showing a 2.9% adverse event occurrence [22]. In 1995, he also quoted a number of studies from the 1950s to the 1990s showing poor agreement among experienced physicians in assessing basic clinical signs at physical examination and in making certain critical diagnoses, such as myocardial infarction [5].

In November 1999, the US Institute of Medicine published a report, *To Err is Human: Building a Safer Health System*, which analysed numerous studies across a variety of organisations, and determined that between 44,000 and 98,000 deaths every year in the USA were the result of preventible medical error [26]. One of the major conclusions was that most medical errors were not the result of individual recklessness or the actions of a particular group, but were most commonly due to faulty systems and processes.

## Categorization of radiologic error

A commonly used and useful delineation divides radiologic error into cognitive and perceptual errors. Cognitive errors, which account for 20–40% of the total, occur when an abnormality is identified but the reporting radiologist fails to correctly understand or report its significance, i.e. misinterpretation. The more common perceptual error (60–80%) occurs when the radiologist fails to identify the abnormality in the first place, but it is recognised as having been visible in retrospect [1]. The reported rate of perceptual error is relatively consistent across many modalities, circumstances and locations, and seems to be a constant product of the complexity of radiologists’ work [1].

In 1992, Renfrew and co-authors classified 182 cases presented at problem case conferences in a US university teaching hospital [27]. The commonest categories were under-reading (the abnormality was missed) and faulty reasoning (including over-reading, misinterpretation, reporting misleading information or limited differential diagnoses). Lesser numbers were caused by complacency, lack of knowledge (the finding was identified but attributed to the wrong cause in both cases) and poor communication (abnormality identified, but intent of report not conveyed to the clinician).

**Fig. 2:**
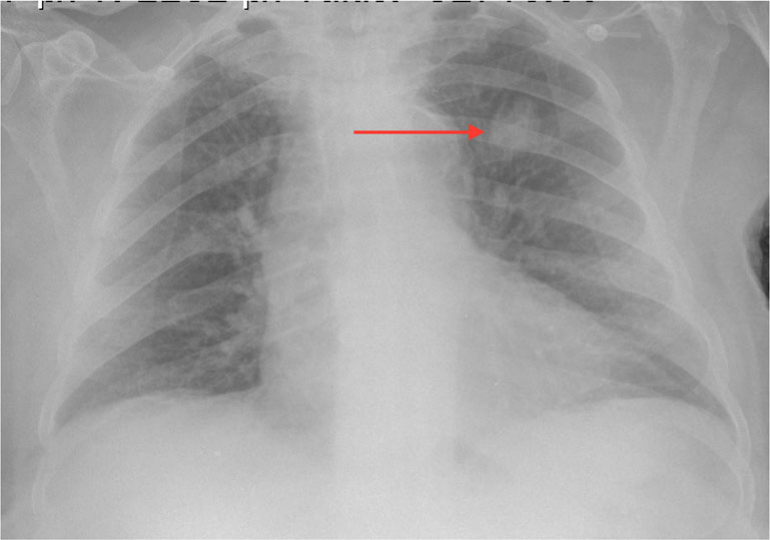
Left upper lobe lung carcinoma (*arrow*), not reported on CXR (under-reading error)

**Fig. 3:**
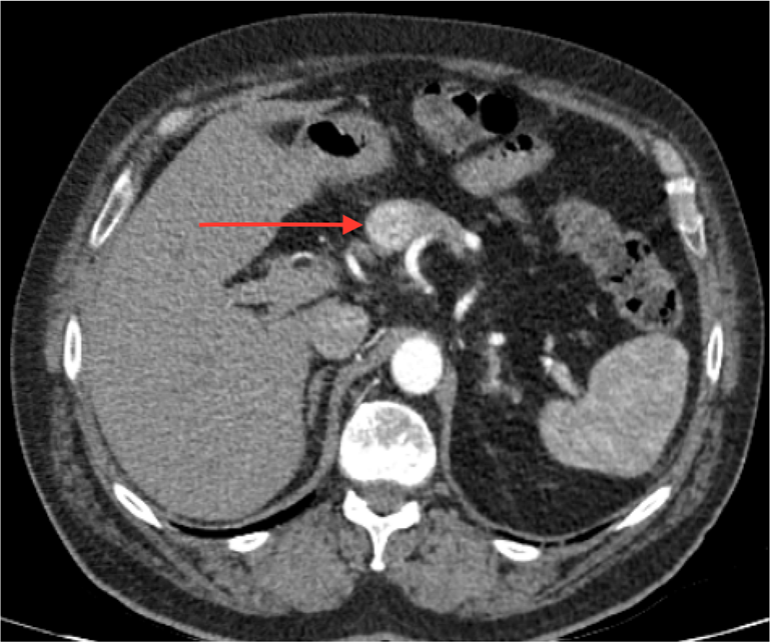
Hypervascular pancreatic metastasis from renal cell carcinoma (*arrow*), not reported on CT; lung and mediastinal nodal metastases identified and reported (satisfaction of search error)

**Fig. 4:**
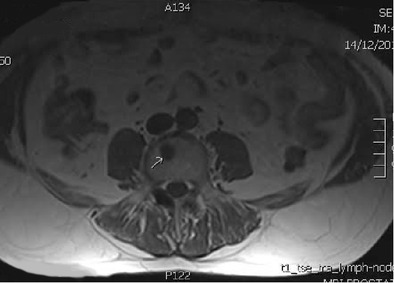
Metastasis from prostate carcinoma (*arrow*), missed on top slice of T1W axial MR sequence (error due to abnormality outside area of interest)

In 2014, Kim and Mansfield published a classification system for radiological errors, adding some useful categories to the Renfrew classification [28, 29]. Their data were derived from 1269 errors (all made by faculty radiologists) reviewed at problem case conferences in a US Army medical centre over an 8-year period. Most errors occurred in plain radiography cases (54%), followed by large-data volume cross-sectional studies: CT 30.5% and MRI 11.4%. The types of errors identified are shown in Table 2. Examples of errors caused by under-reading, satisfaction of search and an abnormality lying outside (or on the margin) of the area of interest are shown in Figs. 2, 3 and 4, respectively.

**Table 2:** Kim & Mansfield radiologic error categorization, 2014 [28]

Error type	Explanation	%
Under-reading	Abnormality visible, but not reported (Fig. 2)	42%
Satisfaction of search	After having identified a first abnormality, radiologist fails to continue to look for additional abnormalities (Fig. 3)	22%
Faulty reasoning	Abnormalities identified, but attributed to wrong cause	9%
Abnormalities outside area of interest (but visible)	Many on first or last image of CT or MR series, suggesting radiologist’s attention not fully engaged at beginning or end of reviewing series (Fig. 4)	7%
Satisfaction of report (alliterative reasoning [29])	Uncritical reliance on previous report in reaching diagnosis, leading to perpetuation of error through consecutive studies	6%
Failure to consult prior imaging studies		5%
Inaccurate or incomplete clinical history		2%
Correct report failing to reach referring clinician		0.08%

### Communication failings

Poorly written or incoherent reports were not identified in either of these studies, but represent another significant source of potential harm to patients. Written reports, forming a permanent part of the patient record, represent the principal means of communication between the reporting radiologist and the referrer. In some instances, direct verbal discussion of findings will take place, but in the vast majority of cases, the radiology report offers the only opportunity for a radiologist to convey his/her interpretation, conclusions and advice to the referrer. However, there can be a considerable difference between the radiologist’s understanding of the message in a radiology report, and the interpretation of that report by the referring clinician [30].

It matters little to a patient if an abnormality is identified by the reporting radiologist and correctly described in the report, if that report is not sufficiently clear for the referring clinician to appreciate what he or she is being told by the radiologist [1]. Among the failings which can lead to misunderstanding of the intent of reports are poor structure or organisation, poor choice of vocabulary, errors in grammar or punctuation, and failure to identify or correct errors introduced into the report by suboptimal voice recognition software. The use of voice recognition software has been found to lead to significantly increased error rates relative to dictation and manual transcription [31], and if the reporting radiologist fails to pay sufficient attention to identifying and correcting such errors, the resulting inaccurate or confusing reports can be a source of significant misunderstanding of the intention of the report by the referrer (a recent example from my own department is the “verified” report of a plain film of the hallux, which includes the phrase “mild metatarsus penis various”—I am assuming this was an uncorrected voice recognition transcription error, as opposed to a description of a foot fetish).

## Factors contributing to radiological error

Technical factors, such as the specific imaging protocol used, the use of appropriate contrast or patient bodily habitus may influence the radiologist’s ability to identify abnormalities or to correctly interpret them [20]. Many possible contributing factors may lead to a radiological error, in the absence of a specific technical explanation, but when identifiable, they can be usefully divided into those that are person (radiologist)-specific, and those that are functions of the environment within which the radiologist works (system issues) [32]. The reporting radiologist may not know enough to identify or recognise the relevant finding (or to correctly dismiss insignificant abnormalities). He may be complacent or apply faulty reasoning. She may consistently over- or under-read abnormalities. He may not communicate his findings or their significance appropriately [27].

Possible system issues leading to error may involve staff shortages and/or excess workload, staff inexperience, inadequate equipment, a less than optimal reporting environment (e.g. poor lighting conditions) or inattention due to constant repetition of similar tasks. Unavailability of previous studies for comparison was a more common contributor in the pre-PACS [picture archiving and communication system] era, but should not be a significant factor in the current digital age. Inadequate clinical information or inappropriate expectations of the capabilities of a radiological technique can lead to misunderstanding or miscommunication between the referring doctor and the radiologist [33]. (The impact of lack of clinical information may be over-estimated, however. In 1997, Tudor evaluated the impact of the availability of clinical information on error rates when reporting plain radiographs. Five experienced radiologists reported a mix of validated normal and abnormal studies 5 months apart, with no clinical information on the first occasion and with relevant clinical information on the second occasion. Mean accuracy improved from 77% without clinical information to 80% on provision of the clinical information, with modest improvements in sensitivity, specificity and inter-observer agreement as well [19].)

Frequent interruptions during the performance of complex tasks such as reporting of cross-sectional studies can lead to loss of concentration and failure to report abnormalities identified but forgotten when the radiologist’s attention was diverted elsewhere. Frequent clinico-radiological contacts have been shown to have a significant positive influence on clinical diagnosis and further patient management; these are best undertaken through formal clinico-radiological conferences [34], but are often informal, and can have a distracting effect when they interfere with other, ongoing work.

Common to all of these system issues is the theme of fatigue, both visual and mental.

Modern healthcare systems frequently demand what has been called hyper-efficient radiology, where almost instantaneous interpretation of large datasets by radiologists is expected, often in patients with multiple co-morbidities, and sometimes for clinicians whose in-depth knowledge of the patients is limited or suboptimal [35]. The pace and pattern of in-hospital care often results in imaging tests being requested before patients have been carefully examined or before detailed histories have been taken. It is hardly surprising that relevant information is not always communicated fully or in a timely manner. There is constant pressure on radiology departments to increase speed and output, often without adequate prior planning of workforce requirements. Error rates in reporting body CT have been shown to increase substantially when the number of cases exceeds a daily threshold of 20 [30]. Many of us feel we are reporting too many studies, too quickly, without adequate time to fully consider our reports. This results in the obvious risk of reduced accuracy in what we report, but also in more unexpected dangers. Berlin reported on a case where a plaintiff claimed that a radiologist’s behaviour in being overworked constituted *“reckless behaviour”,* leading to the radiologist failing to diagnose breast cancer on a screening mammogram, as a result of a *“wanton disregard of patient well-being by sacrificing quality patient care for volume in order to maximise revenue”* [36].

### Workload vs workforce

Data from 2008 [37] show variation in the number of clinical radiologists per 100,000 population in selected European countries, ranging from 3.8 (Ireland) to 18.9 (Denmark). Against this background, the total number of imaging tests performed in virtually all developed countries continues to rise, with the greatest increase in data- and labour-intensive cross-sectional imaging studies (ultrasound, CT and MR). Even within these large-scale figures, there are other, hidden elements of increased workload: between 2007 and 2010, British data demonstrated increases of between 49% and 75% in the number of images presented to the radiologist for review as part of different body part CT examinations [37].

In 2011, a national survey of radiologist workload showed that in 2009, Ireland had approximately two-thirds of the consultant radiologists needed to cope with the workload at the time, applying international norms [38–40]. With increasing workload since that time, and only a modest increase in radiologist numbers, that radiologist shortfall has only worsened.

### Visual fatigue

Krupinski and co-authors measured radiologists’ visual accommodation capability after reporting 60 bone examinations at the beginning and at the end of a day of clinical reporting. At the end of a day’s reporting, they found reduced ability to focus, increased symptoms of fatigue and oculomotor strain, and reduced ability to detect fractures. The decrease in detection rate was greater among residents than attending radiologists. The authors quote conflicting research from the 1970s and 1980s, some of which found a lower rate of detection of lung nodules on chest x-rays at the end of the day, and some which found no change in performance between early and late reporting [41].

### Decision (Mental) fatigue

The length of continuous duty shifts and work hours for many healthcare professionals is much greater than that allowed in other safety-conscious industries, such as transportation or nuclear power [42]. Sleep deprivation has been shown experimentally to produce effects on certain mental tasks equivalent to alcohol intoxication [42]. Continuous prolonged decision-making results in decision fatigue, and the nature of radiologists’ work makes us prone to this effect. Not surprisingly, this form of fatigue increases later in the day, and leads to unconscious taking of shortcuts in cognitive processes, resulting in poor judgement and diagnostic errors. Radiology trainees providing preliminary interpretations during off-hours are especially prone to this effect [43].

###  Inattentional blindness

Inattentional blindness describes the phenomenon wherein observers miss an unexpected but salient event when engaged in a different task. Researchers from the Harvard Visual Attention Lab provided 24 experienced radiologists with a lung nodule detection task. Each radiologist was given five CTs to interpret, each comprising 100–500 images, and each containing an average of ten lung nodules. In the last case, the image of a gorilla (dark, in contrast to bright lung nodules on lung window settings) 48 times larger than the average nodule, was faded in and out close to a nodule over five frames. Twenty of the 24 radiologists did not report seeing the gorilla, despite spending an average of 5.8 s viewing the slices containing the image, and despite visual tracking confirming that 12 of them had looked directly at it [44].

### Dual Process theory of reasoning

The current dominant theoretical model of cognitive processing in real-life decision-making is the dual-process theory of reasoning [43, 45], which postulates type 1 (automatic) and type 2 (more linear and deliberate) processes. In radiology, pattern recognition leading to immediate diagnosis constitutes type 1 processing, while the deliberate reasoning that occurs when the abnormality pattern is not instantly recognised constitutes type 2 reasoning [43]. Dynamic oscillation occurs between these two forms of processing during decision-making.

Both of these types of mental processing are subject to biases and errors, but type 1 processing is especially so, due to the mental shortcuts inherent in the process [43]. A cognitive bias is a replicable pattern in perceptual distortion, inaccurate judgement and illogical interpretation, persistently leading to the same pattern of poor judgement. Type 1 processing is a useful and frequent technique used in radiological interpretation by experienced radiologists, and rather than eliminating it and its inherent biases, the best strategy for minimising these biases may be learning deliberate type 2 forcing strategies to override type 1 thinking where appropriate [43].

### Biases

Many cognitive biases have been described in the psychology and other literature; some of these are particularly likely to feature in faulty radiological thinking, and are listed in Table 3. One might imagine that being aware of potential biases would empower a radiologist to avoid these pitfalls; however, experimental efforts to reduce diagnostic error in specialties other than radiology by applying de-biasing algorithms have been unsuccessful [1].

**Table 3:** Examples of cognitive biases likely to feature in faulty radiological thinking [1, 42]

Bias	Explanation
Anchoring bias	During the process of reporting a study, the radiologist fixes upon an early impression, and fails to adapt or change that view, discounting any subsequent information that may conflict
Framing bias	The radiologist is unduly influenced by the way the question or problem is framed, e.g. if the clinical information provided in a request for a CT states “young patient with palpable mass, probable Crohn’s disease”, a bowel mass may be interpreted as being likely due to Crohn’s, discounting possible malignancy
Availability bias	Tendency to suggest diagnoses that readily come to mind.
Confirmation bias	Tendency to seek evidence to support a diagnostic hypothesis already made, and to ignore evidence refuting that hypothesis
Satisfaction of search	Tendency to stop looking for additional abnormal findings on a study once an initial probable diagnosis is identified
Premature closure	Tendency to accept a diagnosis before proof or verification is obtained
Outcome bias	Naturally empathic inclination to favour a diagnosis that will result in a more favourable outcome for the patient, even if unsupported by evidence
Zebra retreat	Inclination of a radiologist to hold back from making a rare diagnosis due to lack of confidence about reporting such an unusual condition, despite supporting evidence

## Strategies for minimising radiologic error

Many radiologists have traditionally believed that their role in patient care consists in reporting imaging studies. This limited view is no longer tenable, as radiologists have expanded into areas of economic gatekeeping, multidisciplinary team participation, advocacy, and acting as controllers of patient and staff safety. Another role of increasing importance is that of identifying and learning from error and discrepancies, and leading efforts to change systems when systemic issues underpin such errors [46].

The large amount of data available to us leads to the inevitable conclusion that radiological (and other medical) error is inevitable: *“Errors in judgement must occur in the practice of an art which consists largely in balancing probabilities”* [47]. Although it requires a nuanced understanding of the complexity of medical care often not appreciated by patients, politicians or the mass media, acceptance of the concept of necessary fallibility needs to be encouraged; public education can help. Fortunately, many errors identified by retrospective reviews are of little or no significance to patients; conversely, some significant errors are never discovered [3]. The public has a right to expect that all healthcare professionals strive to exceed the appropriate threshold which defines the border between clinically acceptable, competent practice, and negligence or incompetence. Difficulties arise, however, in attempting to identify exactly where that threshold lies.

*Quality management* (or *quality improvement - QI*) in radiology involves the use of systematically collected and analysed data to ensure optimal quality of the service delivered to patients [48]. Increasingly, physician reimbursement for services and maintenance of licensing for practice are being tied to participation in such quality management or improvement activities [48].

Various strategies have been proposed as tools to help reduce the propensity for radiological error; some of these are focused and practical, while others are rather more nebulous and aspirational:

- During the education of radiology trainees (potential error-committers of the future), the inclusion of **meta-awareness** in the curriculum can at least make future independent practitioners aware of limitations and biases to which they are subject but of which they may not have been conscious [43].
- The use of **radiological–pathological correlation** in decision-making, where possible, can avoid some erroneous assumptions, and can ingrain the practice of seeking histological proof of diagnoses before accepting them as incontrovertible.
- Defining **quality metrics**, and encouraging radiologists to contribute to the collation of these metrics and to meet the benchmarks derived therefrom, can promote a culture of questioning and validation. This is the strategy underpinning the Irish national **Radiology Quality Improvement (QI) programme**, operated under the aegis of the Faculty of Radiologists of The Royal College of Surgeons in Ireland [49]. This programme has involved the development and implementation of information technology tools to collect peer review and other QI activities on a countrywide basis through interconnected PACS/radiology information systems (RIS), and to analyse the data centrally, with a view to establishing national benchmarks of QI metrics (e.g. percentage of reports with peer review, prospectively or retrospectively, cases reviewed at QI [formerly discrepancy] meetings, number of instances of communication of unexpected clinically urgent reports, etc.) and encouraging radiology departments to meet those benchmarks. Radiology departments and larger healthcare agencies elsewhere are engaged in similar efforts [50].
- The use of **structured reporting** has been advocated as an error reduction strategy. Certainly, this has value in some types of studies, and has been shown to improve report content, comprehensiveness and clarity in body CT. Furthermore, over 80% of referring clinicians prefer standardised reports, using templates and separate organ system headings [51]. A potential downside to the use of such standardised reports is the risk that unexpected significant findings outside the specific area of clinical concern may be missed by a clinician reading a standardised report under time pressure, and focusing only on the segment of the report that matches the pre-test clinical concern. Careful composition of a report conclusion by the reporting radiologist should minimise this risk.
- Radiologists should pay appropriate attention to the structure, content and language of even those reports where standardised report templates are not being used. With modern PACS/RIS systems using embedded **voice-recognition dictation**, radiologists must take on the task of proofreading and correcting their own dictation, a task many have delegated to transcriptionists in the past. This can be considered as both a contribution to workload and an opportunity: acting as our own proofreaders gives us the facility to tweak our initial dictation to optimise its comprehensibility, and to make reading and understanding it easy. We should embrace this opportunity rather than complaining about the time lost to this activity, and we should ensure that we train our future colleagues in this fundamental task of clear, effective communication.
- The use of **computer-aided detection** certainly has a role in minimising the likelihood of missing some radiologic abnormalities, especially in mammography and lung nodule detection on CT, but carries the negative consequence of the increased sensitivity being accompanied by decreased specificity [43]; radiologist input remains essential to sorting the wheat from the chaff.
- **Accommodative relaxation** (shifting the focal point from near to far, or vice versa) is an effective strategy for reducing visual fatigue, and should be performed at least twice per hour during prolonged radiology reporting [43].
- **Error scoring**: Heretofore, much of the radiology literature on this topic has emphasised identification and scoring of errors [52], and this emphasis has undoubtedly contributed to the understanding of radiology software developers and vendors such that they have put considerable effort into embedding error scoring systems in many QI and PACS/RIS systems [53]. This does not mean that we should be hidebound by these scoring systems. In 2014, the Royal College of Radiologists (RCR) stated that “grading or scoring errors…was unreliable or subjective,….of questionable value, with poor agreement.” They went on to point out that a scoring culture could fuel a blaming culture, and they highlighted the danger of deliberate or malicious misuse of an error scoring system in the pursuit of personal grievances [54]. US experience with RadPeer scoring has been similar, leading to an overemphasis on scoring and underemphasis on commenting, and low compliance with little feedback [55]. Marked variability in inter-rater agreement has been found in the assignment of RadPeer scores to radiological discrepancies [56]. Over time, in response to greater experience with its use, the language and scoring system in RadPeer has been modified [57]. Therefore, the emphasis on considering cases of error or discrepancy is moving away from the assignment of specific scores, and towards fostering a shared learning experience [58].
- **QI (discrepancy) meetings**: Studies have recently shown that the introduction of a virtual platform for QI meetings, allowing radiologists to review cases and submit feedback on a common information technology (IT) platform at a time of their choosing (as opposed to gathering all participants in a room at one time for the meeting), can significantly improve attendance and participation in these exercises, and thus increase available learning [59, 60]. This scenario also removes the potential for negative “point-scoring” by radiologists among one another at meetings requiring participant physical attendance. Presenting a small number of key images (chosen by the meeting convener), as opposed to using the full PACS study file, is a way to reduce the potential for loss of anonymity (of the patient and the reporting radiologist) during QI meetings, while maintaining the meeting focus of the key learning points [61]. Locally adapted models of these meetings may be required in order to ensure maximum radiologist participation and to accommodate those who work exclusively in subspecialty areas or via teleradiology [62]
- The **Swedish eCare Feedback** programme has been running for a number of years, based on extensive double-reporting, identification of cases where disagreement occurs, and collective study of those cases for learning points [30].
- The traditional medical approach to error and perceived underperformance has been to “name, shame and blame”, which is based on the perception that medical mistakes should not be made, and are indicative of personal and professional failure [10, 30, 63]. Inevitably, this approach tends to drive error recognition and reporting underground, with the consequent loss of opportunities for learning and process improvement. A better approach is to adopt a system-centred approach, focusing on identifying what happened, why it happened, and what can be done to prevent it from happening again: the concept of **“root cause analysis”** [64].
- **Hybrids** are possible. In 2012, Hussain et al. published their experience in using a focused peer review process involving a multi-stage review of serious discrepancies identified with RadPeer scoring, which then had the potential to lead to punitive actions being imposed on the reporting radiologist [65].
- Much has been made of the parallels between the **aviation industry** and medicine in error reporting and management, often focusing on the great differences between the two in terms of training, supervision, support and continuous assessment of performance [66]. Larsen elegantly outlines the unhelpfulness of applying RadPeer-type scoring to aviation incidents, and draws the analogy of complacency in allocating a low score to an incident that could still have led to catastrophe: *“[I]t is a question of studying the what, when and how of an event, or to simply focus on the who…..Peer review can either serve as a coach or a judge, but it cannot successfully do both at the same time”* [53]. Certainly, error measurement alone does not lead to improved performance, and error reporting systems are not reliable measures of individual performance [53], but utilising identified cases of error for non-judgemental group learning can facilitate identification of system factors that contribute to errors, and can have a significant role in overall performance improvement.

Interestingly, in those instances where the adjustment of radiologists’ working conditions in an effort to reduce error (limiting fatigue by adjusting work hours, avoiding pressure to maintain work rate, minimising interruptions and distractions) has been studied, these adjustments have had a negligible effect on error reduction (similar to the results of introducing de-biasing algorithms to decision-making in other specialties, as mentioned above) [1].

## Conclusion

A clinician referring a patient for a radiological investigation is generally looking for a number of things in the ensuing radiologist’s report: accuracy and completeness of identification of relevant findings, a coherent opinion regarding the underlying cause of any abnormalities and, where appropriate, guidance on what other investigations may be helpful. The radiologist’s responses to these needs will depend to some extent on the individual; some of us always strive to include the likely correct diagnosis in our reports, but this can sometimes be at the expense of an exhaustive list of differential diagnoses or an incoherent report. Others take the view that it is more helpful to produce a clear report, with good guidance, but accepting that we may be right only some (hopefully most) of the time. The question as to which is the better approach is open to argument; I tend towards the latter view, but taking this approach demands a mutual understanding between referrer and radiologist of our limitations. When I was a trainee, one of the consultants with whom I worked reported normal chest x-rays as “chest: negative”. At the time, I thought this style of reporting was a little sparse. With experience, I’ve come to understand that this brevity captured the essence of the trust needed between a referring doctor and a radiologist. Both sides of the transaction (and the patients in the middle) must understand and accept a certain fallibility, which can never be completely eliminated. The commoditization of radiology and the increasing use of teleradiology services, which militate against the development of relationships between referrers and radiologists, remove some potential opportunities to develop this trust. Of course it is our responsibility to minimise the limitations on our performance where possible; some of the strategies discussed above can help with this. But, fundamentally, the reporting of radiological investigations is not always an exact science; it is more the art of applying scientific knowledge and understanding to a palette of greys, trying to winnow the relevant and important from the insignificant, seeking to ensure the word-picture we create coheres to a clear and accurate whole, and aiming to be careful advisors regarding appropriate next steps. As radiologists, we are sifters of information and artists of communication; these responsibilities must be understood for the imperfect processes they are.

So, in answer to the question posed in this paper’s title, errors/discrepancies in radiology are both inevitable and avoidable. That is, errors will always happen, but some can be avoided, by careful attention to the reasoning processes we use, awareness of potential biases and system issues which can lead to mistakes, and use of any appropriate available strategies to minimise these negative influences. But if we imagine that any strategy can totally eliminate error in radiology, we are fooling both ourselves and the patients who take their guidance from us.
